# Mixed-method longitudinal investigation of sexual and gender-based violence following COVID-19 in South Africa

**DOI:** 10.1136/bmjph-2024-001697

**Published:** 2025-04-15

**Authors:** Miriam Hartmann, Marie C D Stoner, Simone Storey, Danielle Giovenco, Yanga Zembe Zondi, Nontembeko Qwabe, Anna Mia Ekström, Audrey E Pettifor, Linda Gail Bekker, Anna Kågesten

**Affiliations:** 1 Department of Global Public Health, Karolinska Institute, Stockholm, Sweden; 2 RTI International Berkeley Office, Berkeley, California, USA; 3 University of Cape Town Desmond Tutu HIV Centre, Observatory, South Africa; 4 University of KwaZulu-Natal, Durban, South Africa; 5 The University of North Carolina at Chapel Hill Gillings School of Global Public Health, Chapel Hill, North Carolina, USA

**Keywords:** COVID-19, Public Health, Community Health, Violence

## Abstract

**Background:**

Throughout the COVID-19 pandemic, concerns were raised about unintended effects of measures taken to prevent its spread, on sexual and gender-based violence (SGBV). The United Nations called for understanding how national lockdowns put young people at risk of SGBV. This research is particularly needed in contexts such as South Africa, where pre-existing levels of SGBV are high and limited data has been released.

**Methods:**

This mixed-method longitudinal study characterised trajectories of household-level and partner-level SGBV exposure over 6 months, approximately 1 year after the initial COVID-19 lockdown. Utilising group-based trajectory modelling, survey data from 535 male and female participants, ages 13–24 and qualitative insights from 20 in-depth interviews were analysed.

**Results:**

Two trajectory groups emerged for both household-level and partner-level SGBV: (1) groups of participants with consistently low SGBV levels (household: 77.5%; partner: 89.4%) and (2) groups with high baseline levels of SGBV, followed by decreases to moderate levels (household: 22.5%; partner: 10.8%). Characteristics significantly associated with the latter groups included being female, not employed or in school, food insecurity and symptoms of probable common mental disorders. Qualitative data supported these findings and revealed the mitigating role of positive household communication skills, along with potentially unmeasured levels of technology-facilitated partner violence, occurring over phones and social media during lockdown.

**Conclusions:**

Findings should inform the targeting of financial, food and mental health support to those at higher risk of ongoing violence during future times of crises. Further research on technology-facilitated violence should be conducted to better understand its prevalence.

**WHAT IS ALREADY KNOWN ON THIS TOPIC**

- Global cross-sectional research showed a trend in sexual and gender-based violence (SGBV) increasing among adult women during the COVID-19 crisis.
- Only a few studies have longitudinally assessed SGBV among youth in low-income and middle-income countries following COVID-19. These have demonstrated stable trends in SGBV exposure.

**WHAT THIS STUDY ADDS**

- This study adds to the limited evidence of longitudinal patterns of SGBV among youth following COVID-19 lockdowns in sub-Saharan Africa, identifying a population of youth who were at higher risk of SGBV during lockdowns.
- The mixed-methods results demonstrate that the economic consequences of lockdowns contributed to increased stress and tension in households, leading to household violence exposure among youth.
- Youth experienced limited intimate partner violence due to physical restrictions minimising interactions with partners, but qualitative results also show that psychological violence continued over the phone and online.

**HOW THIS STUDY MIGHT AFFECT RESEARCH, PRACTICE OR POLICY**

- Future research with youth should explore the contribution of violence occurring over technology and its impacts on youth mental health.
- Pandemic and crises responses should target economic stress and mental health among vulnerable households.

## Introduction

 Sexual and gender-based violence (SGBV) is a massive public health issue driven by intersecting risk factors across multiple levels of the socioecological model, including individual, interpersonal, community and societal factors.^1^ While gender and economic inequities play a key role at the societal level,^2^ it is also driven by factors which increase vulnerability at the individual and interpersonal levels, such as poor mental health and conflict resolution skills.^3^ Defined as any violent act that is perpetrated against a person’s will and is based on gender norms and unequal power relationships, SGBV takes on many forms and is inclusive of physical, sexual, emotional or financial violence. It can be perpetrated by intimate partners, family members, acquaintances or strangers.^4^ Women and girls tend to be at higher risk than men and boys; however, evidence, particularly among adolescents, can indicate relatively equal levels of violence exposure across sexes, although with differences by form (eg, adolescent girls experiencing greater levels of emotional and sexual violence).^5^ Both experiencing and witnessing SGBV has profound mental health and behavioural consequences for adolescents, including increased risk of depression, anxiety, post-traumatic stress, substance use and future perpetration or victimisation of violence.^6^,^8^

Throughout the COVID-19 pandemic, concerns were raised about unintended social, economic and health-related effects of measures taken to prevent its spread. Impacts on SGBV were of particular concern, and the United Nations called for understanding how school closures, quarantines and national lockdowns put young people, defined as ages 10–24, at risk of SGBV.^9^ While cross-sectional research conducted within low-income and middle-income countries (LMIC) largely suggested that SGBV increased during the COVID-19 pandemic, compared with prior prevalence data,^10^ longitudinal research among this population is still minimal. Only a few studies—among unmarried young people in sub-Saharan Africa—have been published to date, and these present a more nuanced picture of their experiences of violence. For example, although a study in Uganda found that physical SGBV increased during the time of COVID-19, the overall prevalence of SGBV decreased compared with the year prior.^11^ And another study in Kenya found that the prevalence of intimate partner violence (IPV) or non-partner sexual violence remained steady over time.^12^ Additional research is needed to shed further light on the levels and types of SGBV exposures young people faced over this period, and relevant drivers that could be targeted for intervention.

Surprisingly, little research on how COVID-19 impacted SGBV among young people in South Africa exists, despite its high prevalence of violence.^2^ At least one cross-sectional mixed-method study on COVID-19’s impact on youth socioeconomic status and mental health did reveal that, for 15% of youth, COVID led to more violence in their homes.^13^ Youth described how this increase was linked with socioeconomic impacts of the lockdown, such as job losses and food insecurity, which increased stress, tension and mental health concerns. This has been supported by qualitative work with women as well,^14 15^ by one cross-sectional study among youth in South Africa,^16^ and by several studies among youth in other sub-Saharan African contexts.^12 17 18^ These preliminary findings confirm the relevance of using a socioecological model for understanding risk factors to violence during this time period—an approach confirmed in other settings.^19^ Examining trajectories of violence, rather than single-timepoint associations, allows for the identification of distinct risk patterns—such as persistence, escalation or desistance—following initial experiences. This deeper understanding of how violence unfolds over time and its associated risk factors can inform targeted interventions to mitigate harm in future global public health crises.

To fill this gap, we conducted a mixed-method study to garner an in-depth understanding of young people’s exposure to SGBV following the March 2020 COVID-19 lockdown in South Africa. We focused on two specific aims: (1) identifying different trajectories of SGBV exposure and their associated risk factors (quantitative), and (2) understanding young people’s lived experiences of SGBV exposure during and following the lockdown (qualitative). Data were triangulated to reveal a more comprehensive picture of SGBV exposure for young people during this period.

## Methods

### Study design and context

**Figure 1:**
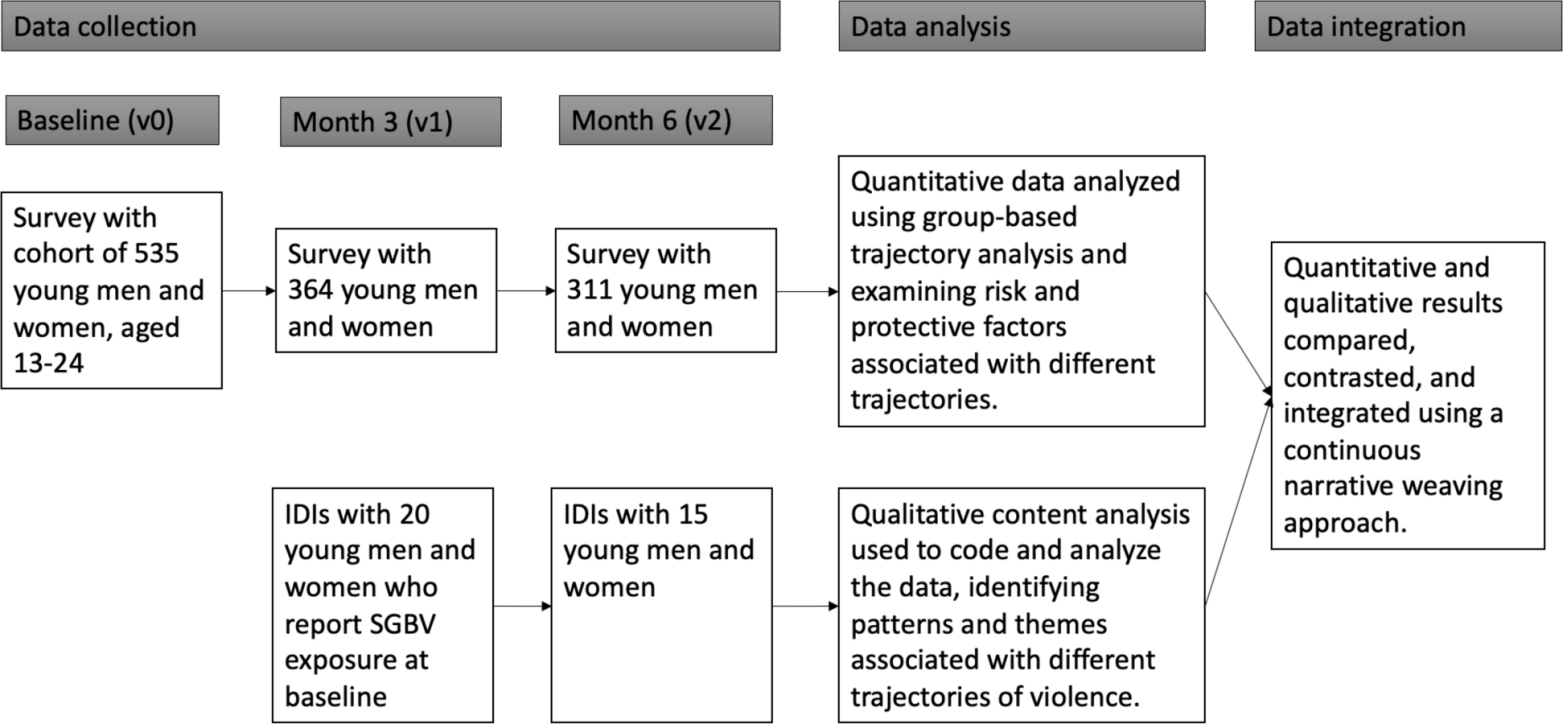
Convergent nested study design. IDIs, in-depth interviews; SGBV, sexual and gender-based violence.

Data were drawn from the BUDDY (Bidirectional, Upbeat communication and Differentiated Distanced care for Young people) study, a mixed-method longitudinal cohort study, which utilised a convergent nested design^20^ where qualitative interviews were conducted within the larger cohort of survey participants (see figure 1). The BUDDY study aimed to both (a) investigate the impact of the COVID-19 lockdown orders on different health-related outcomes including experiences of SGBV among young people living with and without HIV (young people living with HIV, YPLWH and young people living without HIV, YPLWoH), and (b) to test, through a randomised control trial (RCT), the feasibility and acceptability of a remote antiretroviral treatment delivery system among YPLWH. It was conducted in two Cape Town townships within the Cape Flats region, an area that during the Apartheid era was designated for the forced removal of people of colour, and which has been linked to ongoing issues of violence.^21^ More details about the RCT have been described elsewhere.^22^

COVID-19 restrictions in South Africa began on 26 March 2020 and were only fully lifted in January 2022. These restrictions responded to numerous waves of COVID-19 spread, with the most severe restrictions occurring from 26 March to 1 May 2020, when the government enforced a stay-at-home order where individuals could only leave home to purchase food or seek medical care and all sales of alcohol were banned. Further movement was allowed in early May when individuals were allowed to exercise within a 5 km radius of their home between 6 and 9 am. However, curfews continued to restrict freedom of movement until January 2022.^23^

### Participants and procedures

A convenience sample of young people, ages 13–24 years, with regular access to a mobile phone with SMS capacity (for intervention and follow-up purposes), and who resided in the two townships and were not planning to move for at least 6 months were enrolled in the study. Participants were enrolled from February to October 2021 and were followed for 6 months with data collected at baseline, month 3 and month 6. The target sample size for the overall feasibility study was 600. However, a sample size of 352 was deemed sufficient to detect a 15% change/difference in SGBV with 80% power, assuming a baseline prevalence of 30%.^24^ A total of 535 respondents took part in the baseline visit, 434 of these completed the month 3 follow-up (81% response rate) and 240 the month 6 follow-up (45%). Participants not retained were those unable to be contacted at follow-up.

Participants were recruited in two ways, through community outreach teams using flyers and street-based recruitment, and onsite at public clinics or contacted by phone using clinic records. On enrolment, they completed an interviewer-administered baseline survey that assessed sociodemographic characteristics, witnessing SGBV within the household, IPV and variables theorised to be risk or protective factors to SGBV in the context of the COVID-19 pandemic. Variables are described in more detail below and in a baseline manuscript.^16^ Follow-up surveys at month 3 and month 6 asked about experiences in the prior 3 months. The surveys were interviewer-administered telephonically or in-person, depending on participant comfort and COVID-19 restrictions at the time, and data were captured via the secure web-based platform REDcap (Research Electronic Data Capture).^16^

A purposive sample of 20 participants who reported SGBV at baseline were invited to participate in a series of two in-depth interviews (IDIs), occurring after baseline and at month 6, stratified by participant sex (10 males, 10 females) and HIV status (10 YPLWH, 10 YPLWoH). Fifteen of 20 participants completed IDIs at both time points (figure 1). Interviews were conducted by a trained female qualitative interviewer in a private location, using the participant’s chosen language (isiXhosa or English), and were audio-recorded for transcription. Semi-structured guides were used to facilitate discussions on young people’s lived experiences during and after COVID-19 lockdown, including their exposure to various forms of SGBV and discussion of contributing factors at the household and partnership levels. At baseline, they were asked to reflect on their SGBV experiences since the lockdown started, and at month 6, they were asked about the period since their baseline IDI. Audio files were transcribed into English and underwent quality checks to ensure consistency with the audio files.

### Quantitative measures

The primary outcomes were witnessing SGBV among household members and experience of IPV. Household SGBV exposure was a composite measure based on three separate binary questions about witnessing any physical, verbal/emotional or sexual violence in the household at each time point. These three questions represent a condensed version of standard questions asked about SGBV behaviours to reduce administrative burden,^25^ a common practice among studies assessing children’s exposure to parental violence.^26^ An individual was coded as having witnessed household SGBV if they responded ‘yes’ to any of the three items. IPV was assessed at each time point among those reporting having been in an intimate partnership during the queried time point, using an adapted version of the WHO Violence Against Women Instrument,^18^ which asks separately about specific experiences of control, emotional, physical and sexual violent behaviours. Baseline measures asked about exposure since lockdown (for household violence) and during or before lockdown (for IPV). Follow-up measures inquired about experiences in the prior 3 months. See online supplemental file 1 for a table of SGBV measures. A combined binary measure of all forms of IPV was created for each time point where an individual was coded as having experienced IPV if they responded ‘yes’ to any item.

Predictors of interest included sociodemographic factors measured at baseline: age, sex, race, language, household type (formal or informal dwelling), intimate partnership status during lockdown (for household SGBV exposure), school-going status, employment status, hazardous alcohol use and probable common mental disorder (CMD). School and employment status were combined into a measure of ‘not in education, employment or training’ (NEET)^21^ representing participants who were neither in school nor employed. Alcohol use during the past year was measured using the 3-item Alcohol Use Disorders Identification Test (AUDIT-C), with scores >3 for women and >4 for men indicating hazardous alcohol use.^27 28^ Mental health was measured using two validated screening tools based on self-reported symptoms of depression and anxiety. *Depression* was measured using the 9-item Patient Health Questionnaire (PHQ-9), dichotomised to represent the presence of any probable clinical depression defined as a score of 5 and above.^29^ *Anxiety* was measured using the 2-item Generalised Anxiety Disorder (GAD-2), dichotomised to represent the presence of any probable clinical anxiety defined as a score of 3 and above.^30^ Presence of either depression or anxiety was combined into the variable probable *CMD,* where an indication of either depression or anxiety was classified as the presence of a CMD.

### Patient and public involvement

This study involved a youth community advisory board, who were engaged virtually to inform recruitment of the HIV-uninfected participants and to review and provide feedback on all data collection forms. Findings were disseminated to this board in February 2024.

### Analysis

#### Statistical analysis

Group-based trajectory models were used to identify separate trajectories for household SGBV and IPV, respectively, over the 6 month study period. This modelling approach identifies latent groups of participants whose outcomes follow a similar pattern over time^31 32^ and has been used to identify patterns of violence.^33^ These models are helpful to identify heterogeneity within a sample and visualise patterns among groups. We used the *traj* command in Stata to estimate trajectories. *Traj* includes subjects with missing data in the analysis, using only the available data for each subject and maximum likelihood (ML) method, maximising the use of available information. ML estimates model parameters that can then be used to project participant trajectories, even in the presence of missing data, by leveraging patterns in the observed data and filling in gaps with model-based predictions.^34^ For the IPV trajectory models, which include only those participants who reported having a partner at each time point, we conducted a sensitivity analysis where we coded participants without a partner during the set time period as not experiencing IPV. For all analyses, we started by fitting a series of unconditional trajectory models to determine the best number of trajectory groups. Model fit was assessed by comparing the log-likelihood of the model, using Akaike Information Criteria and Bayesian Information Criteria, prioritising smaller values that indicate better fit for these measures, and higher values of entropy and average posterior probability per group, ensuring entropy and posterior probability remained above an acceptable threshold level of social science research.^35^,^38^ Following the selection of the number of groups, we assessed the optimal model shape by testing a range of constant, linear, quadratic and cubic specifications for both trajectory shapes. We selected a final model through further assessment of model fit statistics, as described above, and through visual inspection which involved examining the overlap of CIs and correspondence with observed data points. See online supplemental file 2 for the fit statistics for a selection of models tested. After final model selection, models were adjusted for baseline characteristics that have a theoretical and empirical grounding in their relationship with violence (ie, age, sex, household type, intimate partnership status during lockdown, NEET status, hazardous alcohol use and probable CMD).^1 16^ We assessed multicollinearity using Pearson correlation statistics for all covariates. Correlations between food insecurity, household type and NEET status were all <0.8 and were retained in the model. Mental health was correlated (>0.8) with household type, but both variables were still included because they are important based on the literature, represent different constructs and models still converged and were stable. As a sensitivity analysis, models were also adjusted for the location of intervention (ie, in-person or over the phone); however, this did not result in a meaningful change and was thus removed to avoid overfitting. Models stratified by sex were conducted, but are not presented due to the limited power to converge.

Finally, a variable was then created and assigned to an individual based on the highest probability of trajectory group membership. This classification was used to describe membership in relation to baseline characteristics of age, sex, household type, intimate partnership status during lockdown, NEET status, hazardous alcohol use and probable CMD. We also compared membership by number of study visits completed to assess whether loss to follow-up may have impacted assignment and biased results.^39^ T-tests for continuous variables (eg, age) and χ^2^ tests for most binary variables were examined to identify differences in baseline covariates by trajectory membership. STATA V.17 was used for statistical analyses.^40^

#### Qualitative analysis

The qualitative interviews were analysed using content analysis, a method that is suitable to interpret, classify and code data according to themes and patterns.^41^ Using the qualitative analysis software Dedoose, two analysts (one from the USA and one from South Africa) coded all transcripts using a codebook that was developed following review of an initial set of transcripts and refined throughout the coding process. Codes included those that captured trajectories of violence based on participant descriptions of patterns in their experience of violence such as improvements, sustainment and worsening situations. These were subsequently coded as ‘decreased’, ‘stayed the same’ or ‘increased’. They also included factors associated with described violence such as ‘substance use’, ‘fidelity’ and ‘unemployment’. Several methods were used to ensure intercoder reliability, including co-coding 20% of the transcripts and discussing discrepancies to reach consensus. Weekly meetings and periodic coding tests in Dedoose were also used to maintain coding reliability.

Code reports were stratified by participant sex, type of violence (ie, household or partner) and by participant identified patterns in violence (decreased, stayed the same or increased). Themes were identified around the contextual experience of SGBV, for example, experiences of food insecurity, unemployment, stress and mental health, and how and why they are described as impacting SGBV, if at all, and how and why either of these factors changed over time.

#### Mixed-method analysis

We used an *embedding* approach (a combination of *building* and *merging*) for our mixed-methods analysis, as described by Fetters *et al*.^20^ This involved our quantitative data collection informing our qualitative coding, and the merging of data in analysis for comparison. As our quantitative survey was designed to assess SGBV in different spheres (ie, intimate partnerships and in the household), and known drivers of SGBV at the individual, interpersonal and structural levels, we thus developed qualitative codes to capture discussions of intimate partnerships and households, as well as for specific drivers of violence such as emotions and mental health, relationship features and economic stressors. Following initial separate analyses, a common approach in convergent nested designs,^20^ results were then merged according to topic area. This was done by looking at quantitative and qualitative findings within each relationship sphere, comparing, for example, quantitative trends in violence with qualitative descriptions and comparing quantitative associations with what was discussed qualitatively as contributing to violence. In these comparisons, we looked for areas of agreement, disagreement and how the qualitative data may explain our quantitative findings. Using a narrative weaving approach, we jointly present quantitative and qualitative results according to violence sphere and identified drivers.

#### Ethical considerations

Both the University of Cape Town Human Research Ethics Committee (HREC REF: 448/2020), the local ethics committee where one study PI is based (LGB) and the Swedish Ethical Review Authority (EPN Dnr 2020-04903) where collaborating PIs are based (AME, AK) reviewed and approved the research study’s protocol and procedures. Written informed consent or assent (if under 18 years) was obtained from all research participants prior to enrolment. Participants under 18 had the option of either obtaining or waiving parental consent. Additional ethical and safety protocols were also developed in order to respond to disclosures of violence, which followed mandatory reporting guidelines according to the Children’s Act of 2005 in South Africa.^42^ Procedures were implemented to ensure that all participants reporting violence were offered a connection to a study counsellor, followed by referral to a Social Worker for ongoing care. This was mandated for those under 18, such that study staff brought participants to the Social Worker for care. Finally, the research team also monitored social harms throughout the study to identify and respond to any instances of abuse as a result of the study.

## Results

### Participant characteristics

At baseline, participants were on average 19.1 years of age. The majority were female (70%), Black African (99.4%) and isiXhosa speaking (97.9%). About four in 10 (40.2%) reported living with HIV as per the design of the broader BUDDY study. Most participants lived in formal dwellings (61.9%) with an average of 4.2 household members. About half had an intimate partner at the time of the COVID-19 lockdown (49.8%). Approximately one-third were not employed or in school (31.7%). Over half could be classified as food insecure (51.5%), and as engaging in hazardous drinking (56.4%). Just under half reported symptoms of probable CMD (46.9%). See table 1. Notably, almost no participants lived with their partner (n=2; 0.4%) (not shown).

**Table 1:** Baseline participant characteristics, overall and by household SGBV and IPV trajectory groups

Baseline variables	Total,N (%)535	Household SGBV trajectory group			IPV trajectory group		
		Group 1: consistently low,n (%)412 (77%)	Group 2: declining to moderate,n (%)123 (23.0%)	P value	Group 1: consistently low, n (%)467 (87.3%)	Group 2: declining to moderate,n (%)68 (12.7%)	P value
Visits completed				0.074			0.21
Baseline only	101 (18.9)	83 (20.1)	18 (14.6)		89 (19.1)	12 (17.6)	
Baseline and M3-only	194 (36.3)	155 (37.6)	39 (31.7)		175 (37.5)	19 (27.9)	
Baseline, M3 and M6	240 (44.9)	174 (42.2)	66 (53.7)		203 (43.5)	37 (54.4)	
Current age, mean (SD)	19.1 (3.0)	18.8 (3.1)	19.7 (2.8)	0.005	18.8 (3.0)	20.5 (2.8)	<0.001
Sex				0.011			<0.001
Female	373 (69.7)	295 (71.6)	78 (63.4)		334 (71.5)	39 (57.4)	
Male	160 (29.9)	117 (28.4)	43 (35.0)		133 (28.5)	27 (39.7)	
HIV status				<0.001			0.001
YPLWH	215 (40.2)	186 (45.1)	29 (23.6)		200 (42.8)	15 (22.1)	
YPLWoH	320 (59.8)	226 (54.9)	94 (76.4)		267 (57.2)	53 (77.9)	
Black African race	532 (99.4)	411 (99.8)	121 (98.4)	0.41	465 (99.6)	67 (98.5)	1.0
IsiXhosa speaking	524 (97.9)	404 (98.1)	120 (97.6)	0.85	458 (98.1)	66 (97.1)	1.0
Household type				0.97			<0.001
Formal dwelling	331 (61.9)	255 (61.9)	76 (61.8)		302 (64.7)	29 (42.6)	
Informal dwelling	198 (37.0)	154 (37.4)	44 (35.8)		163 (34.9)	35 (51.5)	
No. of people in household, mean (SD)	4.2 (2.6)	4.2 (2.5)	4.3 (3.1)	0.80	4.3 (2.6)	3.9 (3.0)	0.26
Had a sexual partner during lockdown	262 (49.8)	200 (48.5)	62 (54.4)	0.27			
NEET	168 (31.7)	114 (27.7)	54 (45.8)	<0.001	221 (47.4)	43 (68.3)	<0.001
Food insecure	274 (51.5)	191 (46.4)	83 (69.2)	<0.001	125 (26.8)	47 (72.3)	<0.001
Hazardous drinking (AUDIT-C)	181 (56.4)	133 (55.6)	48 (58.5)	0.65	227 (48.6)	27 (57.4)	0.87
Probable CMD	247 (46.9)	147 (35.7)	100 (87.0)	<0.001	154 (56.2)	54 (90.0)	<0.001

P-values based on t-test, Pearson’s χ2 test and Fisher’s exact test.

Scores on PHQ-9 range from 1 to 27 with scores ≥5 indicating at least mild depression. Scores≥3 on GAD-2 indicate generalised anxiety. Common mental disorders refer to the presence of clinically relevant depression or anxiety.

Two participants (0.4%) selected ‘other’ gender, 1 (0.2%) selected ‘coloured’ race, 1 (0.2%) selected ‘white’ race, 3 (0.6%) selected ‘Afrikaans’ as primary language and 4 (0.8%) selected ‘other’ household type and two did not answer this question.

Trajectory groups models were adjusted for gender, HIV status, household composition (no. of people in the household), food insecurity and probable CMD.

AUDIT-C, Alcohol Use Disorders Identification Test; CMD, common mental disorder and includes measures of probable depression or anxiety based on non-clinical screening; IPV, intimate partner violence; M3, month 3 visit; M6, month 6 visit; NEET, not in education, employment or training; PLWoH, young people living without HIV; SGBV, sexual and gender-based violence; YPLWH, young people living with HIV.

#### Overall exposure to SGBV

At baseline, approximately one-third of participants reported witnessing any violence in their households (32.8%), which was largely driven by witnessing emotional (23%) or physical (19.8%) violence. Thirteen per cent of participants experienced IPV (13.6%) at baseline, with the highest proportion of these reporting exposure to physical violence from their partner (8.4%). See online supplemental file 3.

#### Trajectories and experiences of household SGBV exposure

**Figure 2:**
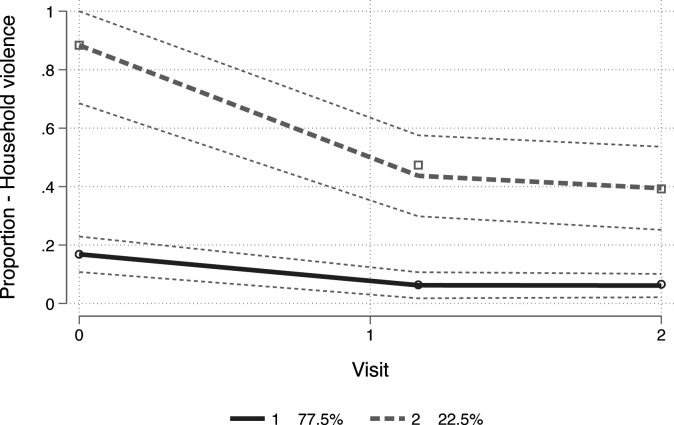
Trajectories of household sexual and gender-based violence exposure. 1, as trajectory group with consistently low exposure from baseline to month 6 (77.5% of the study sample) (‘consistently low’); 2, a trajectory group with an initial large drop in exposure from baseline to month 3 that was maintained from months 3 to 6 (22.5% of the study sample) (‘declining to moderate’).

Participants fell into two trajectory groups (ie, segments of the study population), in the adjusted quantitative model, in terms of household SGBV exposure: (1) a group with consistently low exposure from baseline to month 6 (77.5% of the sample) (‘consistently low’), and (2) a group with an initial large drop in exposure from baseline to month 3 that was maintained from months 3 to 6 (22.5%) (‘declining to moderate’) (Bayesian information criterion, BIC=−577.65; entropy 0.692). See figure 2 for the two trajectory groups and their associated study sample proportions. Ninety per cent of group 2 reported exposure at baseline, dropping to 45% at month 3 and 40% at month 6. See online supplemental file 3. In terms of associated baseline characteristics, being older (p=0.005), female (63.4%; p=0.011), living without HIV (76.4%; p<0.001), NEET status (45.8%, p<0.001), being food insecure (69.2%; p<0.001) and symptoms of a probable CMD (87.0%; p<0.001) were significantly associated with membership in the ‘declining to moderate’ trajectory group. Number of follow-up visits completed was not associated with membership. See table 1.

Participant experiences described in the qualitative data collection revealed several intersecting themes related to the physical, social and emotional impacts of COVID-19 on household relationship dynamics. These included the role of physical *COVID-19 restrictions*, such as quarantining and alcohol bans, on relationships, as well as the physical and emotional impact of *financial strain and food insecurity. Communication and conflict negotiation* patterns intersected with these shared experiences during COVID-19 lockdown, but also were framed as more static characteristics of household social bonds.

##### COVID-19 restrictions—quarantining and alcohol bans

While many participants described negative social emotional experiences associated with quarantining, primarily via the impact of school closures, confinement to one’s home was not linked to household conflict or violence. A few participants, two males and one female, spoke about how spending time with one’s family under quarantine improved familial relationships and communication. One male participant said this helped him feel supported when depressed. A female participant spoke of how everyone contributed to the family via household chores, which reduced strain. Finally, another male participant explained that familial relations were improved by reductions in alcohol use.

Interviewee: During weekends, they will not listen to you as a child but now they care about us…We help each other. I am able to sit down with my parent and discuss my challenges. I cannot say it was difficult before but it was not happening.

Interviewer: So COVID helped a lot.

Interviewee: It helped a lot.

Interviewer: It reduced the intake of beer because parents could spend time with their children.

Interviewee: Yes. (Male, YPLWH, Baseline)

##### Financial strain and food insecurity

Supporting quantitative findings showing a relationship between household violence and financial strain and food insecurity, a second major theme was the role of these experiences in increasing conflict and violence in the household. A couple of participants noted that government social relief cash transfers supported their families, but this was not always enough, as one female participant said: *‘*It was difficult, the grant money was not enough to buy food’ (Female, YPLWoH, Baseline). And another participant described how her uncle, whom she was living with, began to physically abuse her aunt now that her aunt was unemployed and asking for more financial support from him.

Interviewee: It was just happening during that time, before lockdown he was not beating [her] when she was still working. This beating is new.

Interviewer: It is because of this lockdown?

Interviewee: Yes, it is because of lockdown.

Interviewer: So, this fighting between starts because of money or there are other reasons?

Interviewee: Maybe there are other issues that I personally do not know ……but when I see it, it is this thing of money. (Female, YPLWoH, Baseline)

Notably, for one female participant, the economic strain, which was described as an ongoing issue, created coalescence across family members during COVID because everyone understood and accepted it during this time, which led to cooperation rather than conflict. Relatedly, participants also described how (re-)gained employment and receipt of cash transfers at follow-up reduced conflict in the home.

### Trajectories and experiences of IPV exposure

**Figure 3:**
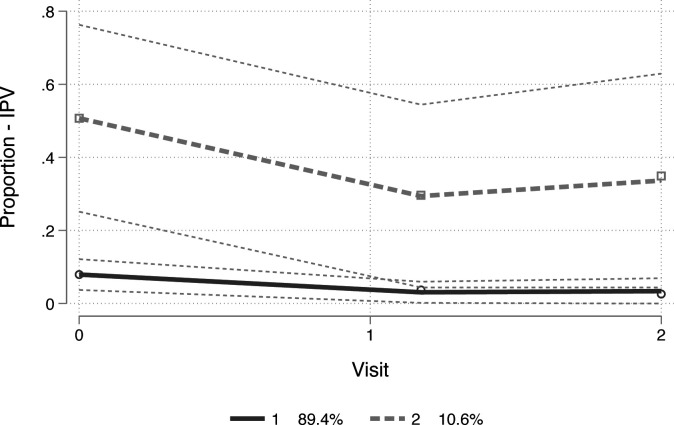
Trajectories of intimate partner violence exposure. 1, trajectory group with no/low IPV exposure referred to as ‘consistently low’ (89.4% of the study sample); 2, a s trajectory group where IPV dropped from baseline to month 3, but slightly increased from months 3 to 6 referred to as ‘declining to moderate’ (10.6% of the study sample). IPV, intimate partner violence.

Similar to household SGBV exposure, participants fell into two quantitative trajectory groups in the adjusted model, in terms of IPV exposure. These included (1) one group with no/low IPV exposure (89.4% of the sample) (‘consistently low’) and (2) a second group where IPV dropped from baseline to month 3, but slightly increased from months 3 to 6 (10.6%)(‘declining to moderate’) (BIC=−197.16; entropy 0.843). See figure 3 for the two trajectory groups and their associated study sample proportions. Approximately 50% of group 2 reported exposure to IPV at baseline, followed by 31% at month 3 and 37% at month 6. See online supplemental file 3. Characteristics significantly associated with membership in the ‘declining to moderate’ trajectory group included being older (p<0.001), being female (58.2%; p<0.001), living without HIV (79.1%; p=0.001), NEET status (68.3%; p<0.001), food insecurity status (72.3%; p<0.001) and reporting symptoms of a probable CMD (90.0%; p<0.001). Number of follow-up visits completed was not associated with membership. See table 1.

Participants’ descriptions of IPV corresponded with quantitative findings showing relatively low levels of physical and sexual IPV at baseline. These were primarily due to physical restrictions limiting their ability to visit their partners. As one female participant stated, ‘It was not happening all the time because we were not meeting regularly’ (Female, YPLWoH, Baseline). Qualitative descriptions, however, added depth to understanding what was occurring in terms of ongoing conflict with partners that was likely not captured in quantitative IPV measures. This theme centred around emotional trust and elucidated a shift to conflict over the phone/social media.

#### Mistrust as a result of distance

For some participants, while not described as violence, the distance from their partner enhanced a sense of mistrust, which, combined with a lot of free time to worry, exacerbated concerns about infidelity and subsequently led to conflict.

We had a fight because he was thinking that I am cheating since he is that side. (Female, YPLWH, Follow-up)

It happened when COVID-19 was starting, when we were not supposed to go outside. She always wanted me to be outside to see her. I would hardly go outside so she was always thinking that I am with another girlfriend. (Male, YPLWoH, Baseline)

This theme, however, was described as a common characteristic of many relationships and as an ongoing trigger of conflict that sometimes resulted in violence after the lockdown ceased.

#### Technology-facilitated conflict and violence

Notably, the topic of having conflict over the phone and using phones/technology to control one’s partner came up among both male and female participants. Several men and women described how conflicts moved to the phone during COVID-19 restrictions. One man explicitly said having conflict over the phone could lead to him physically abusing his partner when they saw each other next, while one woman said conflict on the phone felt ‘safer’ as she felt it protected her from the risk of physical violence resulting from in-person conflict.

Interviewee: Maybe, the problem is he has never beaten me, but I always think [if] I ask him when he is close to me…and then he gets angry and wants to beat me. So that is why I don’t ask, I only ask over the phone.

Interviewer: So, you chose to talk to him over the phone and address/resolve your issues over the phone…you are saying you are scared, like is it his anger that makes you to ask him or address things over the phone?…

Interviewee: Yes, it is. (Female, YPLWoH, Baseline)

Conflict over the phone intersected with mistrust, particularly when partners refused to be open about who was calling them or who they interacted with. The phone also became a tool of violence and control for some. Men were primarily the ones described, by males and females alike, as (1) being angered if their female partner did not answer her phone or did not have it on all the time, and (2) checking the female partner’s phones/social media accounts to look for signs of cheating. Only one male partner said his female partner wanted to check his phone, and one female partner said she wanted to know who was calling her partner.

Like he likes to look at my phone and he will see my Facebook friends and he will think that I am in a relationship with them and…he will be angry that I am in love with so and so and he will angry and shout like those things. And I will be like eyi! I am not in love with this person if you don’t want him I can block him. I have blocked my friends because of him. (Female, YPLWH, Baseline)

## Discussion

This longitudinal research study on experiences of SGBV among young people living in peri-urban Cape Town, South Africa, adds to the limited evidence we have in terms of patterns and drivers of SGBV exposure following COVID-19 lockdowns.^11 12 43^ We found that although most young people in our study experienced relatively low levels of SGBV at both the household and partner levels, there were many who faced high levels of violence at the time of the lockdown, followed by decreases in the subsequent 6 months. These youth tended to be out of school or unemployed, facing food insecurity and having symptoms of CMD, aligning with the established socioecological framework for understanding drivers of violence.^1 19^ Qualitatively, participants described periods of high stress and tension in their households and concurred that food insecurity played a strong role in household SGBV. Other dynamics such as communication patterns and trust also influenced these experiences. Conflict with intimate partners over technology, such as phones and social media, was frequently noted and likely not captured by the quantitative trajectories.

Similar to prior literature, our findings highlighted the substantial contribution of the economic consequences of COVID-19 lockdowns to household conflict and violence.^1044^,^48^ Data from South Africa shows that COVID-19 restrictions had a severe impact on the country’s economy and individual economic vulnerability.^49 50^ These impacts on employment and access to financial resources clearly affected resource security, such as food insecurity, further enhancing stress. In line with our findings, the socioeconomic consequences of the lockdowns may have diminished over time as restrictions lessened and as government social distress programmes reached vulnerable individuals.^51^ Participants also described in certain situations how restrictions and constraints could serve to mobilise familial support. This seemed to most often be the case when communication skills were strong, pointing to the need for interventions that strengthen family communication, but also financial and food provisions to reduce stress during times of crisis.

South Africa was unique in their severe restriction of alcohol sales during various phases of COVID-19 restrictions. At numerous points in the pandemic, alcohol sales were banned. These bans have been linked to reductions in trauma in hospitals,^52^ and given the relationship between alcohol use and SGBV,^353^,^55^ one could expect a similar drop in SGBV. Our qualitative research did reveal that young people perceived a drop in violence because of these bans; however, quantitative findings suggested that there were still high levels of alcohol consumption, even at baseline, with more than half of youth in our study exhibiting hazardous drinking behaviours. Yet, drinking was not associated with membership to higher SGBV exposure trajectories. This may be because our measure of alcohol use was among SGBV victims, rather than the perpetrators. Further work should be done to test alcohol-related interventions on SGBV prevention.^56^,^59^

One interesting finding from this study is that IPV among young people in this peri-urban township may have shifted to occur over the phone during the COVID-19 lockdown. Although this type of technology-facilitated SGBV (tf-SGBV) has been a growing topic in the field of SGBV for several years,^60^,^67^ limited research has occurred in South Africa. Research in other settings indicates that exposure to tf-SGBV begins at an early age and may be associated with poor mental health and other psychosocial outcomes.^60 62 64 68 69^ Despite high levels of income inequality in the country, mobile phone penetration and access to online content is widespread in South Africa, including among young people.^70 71^ As a result, our quantitative findings, which showed low levels of IPV compared with South African statistics in non-COVID-19 periods, may not have fully captured young people’s IPV exposures. Future research should incorporate measures of tf-SGBV to ensure a full range of exposures are captured and to better understand its association with health outcomes.^66^

No research is without limitations, and this study, which was designed to rapidly capture trends in SGBV exposure during COVID-19 lockdowns, could have improved data collection methods, retention and measures. Due to COVID-19 restrictions on movement, the baseline data collection was delayed, and it was necessary to collect data through a combination of in-person and telephonic surveys which could have resulted in differential response bias. While there is mixed evidence regarding data quality when conducting surveys on sensitive topics, such as violence over the phone^72^ and lack of privacy, which could have influenced responses related to household SGBV exposure, the research team attempted to ensure privacy and safety by confirming privacy before starting the survey and forewarning participants about upcoming sensitive topics in the survey. The relatively high rates of household violence captured suggest that these strategies may have successfully supported participant reporting. Participant retention, which greatly reduced the sample size from baseline to month 6, is also a limitation. By month 6, about 45% of the sample was retained, which limited the study power. Retention in studies on sensitive topics such as SGBV is a common concern,^73^ and in our case, poor retention was mainly due to the inability to recontact participants, something that was made more difficult due to lockdown movement restrictions and which protracted baseline study enrolment. However, this attrition is in line with two other longitudinal studies published from this time period on adolescent exposure to violence,^11 12^ exceeding 36% retention in the study by Decker *et al*.^12^ The use of trajectory modelling, which applies a ML approach to predicting trajectories with missing data, helped overcome the issue of poor retention, and the fact that completion of all study visits was not significantly different by trajectory group suggests that bias may have been limited. However, the sample size may still have impacted the stability of parameter estimates given the relatively small numbers of participants in the ‘declining to moderate’ groups. While there are no standards for minimum group sizes, ours do meet the standards outlined by Nagin of a minimum of 20–30 participants.^31^ Small groups can limit statistical power to detect differences with baseline characteristics; however, our results found several significant associations suggesting that these groups are distinguishable and not an artefact of model instability. Finally, the study’s measures on exposure to household SGBV could have been strengthened through the addition of questions on who the participant witnessed experiencing the SGBV. Without this information, we are unable to confirm that these experiences were not between siblings, for example, as opposed to other adult family members. Regardless, exposure to violence in the household has severe consequences for individual well-being and is a known risk factor for ongoing cycles of violence in other spheres of one’s life.^74 75^ Despite these limitations, many of which included unique challenges due to the pandemic, this research offers important learning opportunities from a research and implementation perspective, and the triangulation offered through our use of mixed methods strengthens the quantitative trends.

## Conclusion

This research contributes rare and valuable longitudinal data on SGBV among youth during and after the COVID-19 lockdowns in LMIC settings. It appears that an important minority of young people were exposed to high levels of household and partner SGBV during lockdown, followed by a decrease to more moderate, although unacceptable levels of ongoing violence. Exposure was notably related to economic hardship enhanced by the pandemic lockdowns, associated stress and poor communication patterns. Lower levels of IPV may be underestimating true exposure to all forms of IPV, evidenced by qualitative reports of this occurring over technology. Future research should further investigate trends in shifting IPV from in-person to online spaces, and the effect of government cash and food support on mitigating the role of financial hardship on SGBV. Interventions targeting familial stress and communication skills may reduce SGBV in future times of public health crises such as the COVID-19 pandemic.

## Supplementary material

10.1136/bmjph-2024-001697 online supplemental file 1

10.1136/bmjph-2024-001697 online supplemental file 2

10.1136/bmjph-2024-001697 online supplemental file 3

## Data Availability

Data are available upon reasonable request.

## References

[1] Heise LL (1998). Violence against women: an integrated, ecological framework. Violence Against Women.

[2] National Strategic Plan on Gender-based Violence & Femicide (GBVF-NSP) (2020). Johannesburg interim steering committee (ISC) on GBVF.

[3] Gibbs A, Dunkle K, Ramsoomar L (2020). New learnings on drivers of men’s physical and/or sexual violence against their female partners, and women’s experiences of this, and the implications for prevention interventions. Glob Health Action.

[4] UNHCR Gender-based violence. https://www.unhcr.org/gender-based-violence.html.

[5] Meinck F, Cluver LD, Boyes ME (2016). Physical, emotional and sexual adolescent abuse victimisation in South Africa: prevalence, incidence, perpetrators and locations. J Epidemiol Community Health.

[6] Jagasia E, Bloom I, Nelson KE (2024). Early adolescent development in the face of violence: A systematic review running. Child Abuse Negl.

[7] Nicodimos S, Gelaye BS, Williams MA (2009). Associations between witnessing parental violence and experiencing symptoms of depression among college students. East Afr J Public Health.

[8] Sui X, Massar K, Kessels LTE (2021). Violence Exposure in South African Adolescents: Differential and Cumulative Effects on Psychological Functioning. J Interpers Violence.

[9] UNFPA (2020). Impact of the covid-19 pandemic on family planning and ending gender-based violence, female genital mutilation and child marriage. https://www.unfpa.org/resources/impact-covid-19-pandemic-family-planning-and-ending-gender-based-violence-female-genital.

[10] Bourgault S, Peterman A, O’Donnell M (2021). Violence against women and children during covid-19—one year on and 100 papers in: a fourth research round up.

[11] Mayanja Y, Kamacooko O, Lunkuse JF (2023). Prevalence, Perpetrators, and Factors Associated With Intimate Partner Violence Among Adolescents Living in Urban Slums of Kampala, Uganda. J Interpers Violence.

[12] Decker MR, Bevilacqua K, Wood SN (2022). Gender-based violence during COVID-19 among adolescent girls and young women in Nairobi, Kenya: a mixed-methods prospective study over 18 months. BMJ Glob Health.

[13] Duby Z, Bunce B, Fowler C (2022). Intersections between COVID-19 and socio-economic mental health stressors in the lives of South African adolescent girls and young women. Child Adolesc Psychiatry Ment Health.

[14] Dekel B, Abrahams N (2021). “I will rather be killed by corona than by him…”: Experiences of abused women seeking shelter during South Africa’s COVID-19 lockdown. PLoS One.

[15] Mahlangu P, Gibbs A, Shai N (2022). Impact of COVID-19 lockdown and link to women and children’s experiences of violence in the home in South Africa. BMC Public Health.

[16] Hartmann M, Giovenco D, Zeebari Z (2023). Associations between psychosocial wellbeing and experience of gender-based violence at community, household, and intimate-partner levels among a cross-sectional cohort of young people living with and without HIV during COVID-19 in Cape Town, South Africa. BMC Public Health.

[17] Decker MR, Wood SN, Thiongo M (2021). Gendered health, economic, social and safety impact of COVID-19 on adolescents and young adults in Nairobi, Kenya. PLoS One.

[18] Karp C, Moreau C, Sheehy G (2021). Youth Relationships in the Era of COVID-19: A Mixed-Methods Study Among Adolescent Girls and Young Women in Kenya. J Adolesc Health.

[19] Rieger A, Blackburn AM, Bystrynski JB (2022). The impact of the COVID-19 pandemic on gender-based violence in the United States: Framework and policy recommendations. *Psychol Trauma*.

[20] Fetters MD, Curry LA, Creswell JW (2013). Achieving integration in mixed methods designs-principles and practices. Health Serv Res.

[21] Van der Westhuizen M, Gawulayo S (2021). Youths in gangs on the Cape Flats: if not in gangs, then what?. Social Work.

[22] Giovenco D, Pettifor A, Itzikowitz G (2023). Access to sexual and reproductive health services among South African young people living with and without HIV during the COVID-19 pandemic. *Contraception*.

[23] Haider N, Osman AY, Gadzekpo A (2020). Lockdown measures in response to COVID-19 in nine sub-Saharan African countries. BMJ Glob Health.

[24] Dean AG, Sullivan KM, Soe MM (2013). OpenEpi: open source epidemiologic statistics for public health, Version 3.1. https://www.openepi.com/SampleSize/SSCohort.htm.

[25] García-Moreno C, Jansen HA, Ellsberg M (2005). WHO multi-country study on women’s health and domestic violence against women.

[26] Latzman NE, Vivolo-Kantor AM, Clinton-Sherrod AM (2017). Children’s exposure to intimate partner violence: A systematic review of measurement strategies. Aggress Violent Behav.

[27] Morojele NK, Nkosi S, Kekwaletswe CT (2017). Utility of Brief Versions of the Alcohol Use Disorders Identification Test (AUDIT) to Identify Excessive Drinking Among Patients in HIV Care in South Africa. J Stud Alcohol Drugs.

[28] Bush K, Kivlahan DR, McDonell MB (1998). The AUDIT Alcohol Consumption Questions (AUDIT-C): An Effective Brief Screening Test for Problem Drinking. Arch Intern Med.

[29] Kroenke K, Spitzer RL, Williams JB (2001). The PHQ-9: validity of a brief depression severity measure. J Gen Intern Med.

[30] Bhana A, Mntambo N, Gigaba SG (2019). Validation of a brief mental health screening tool for common mental disorders in primary healthcare. *S Afr Med J*.

[31] Nagin DS (2014). Group-based trajectory modeling: an overview. Ann Nutr Metab.

[32] Nagin DS, Jones BL, Passos VL (2018). Group-based multi-trajectory modeling. Stat Methods Med Res.

[33] Wojciechowski TW (2020). PTSD as a Risk Factor for the Development of Violence Among Juvenile Offenders: A Group-Based Trajectory Modeling Approach. J Interpers Violence.

[34] Carpenter JR, Smuk M (2021). Missing data: A statistical framework for practice. *Biometrical J*.

[35] Mésidor M, Rousseau M-C, O’Loughlin J (2022). Does group-based trajectory modeling estimate spurious trajectories?. BMC Med Res Methodol.

[36] Nagin DS, Odgers CL (2010). Group-based trajectory modeling in clinical research. Annu Rev Clin Psychol.

[37] Spurk D, Hirschi A, Wang M (2020). Latent profile analysis: A review and “how to” guide of its application within vocational behavior research. J Vocat Behav.

[38] Muthén B (2004). The Sage handbook of quantitative methodology for the social sciences, 345.

[39] Haviland AM, Jones BL, Nagin DS (2011). Group-based Trajectory Modeling Extended to Account for Nonrandom Participant Attrition. Sociol Methods Res.

[40] StataCorp (2021). Stata statistical software: release 17.

[41] Braun V, Clarke V (2021). Can I use TA? Should I use TA? Should I *not* use TA? Comparing reflexive thematic analysis and other pattern‐based qualitative analytic approaches. Couns and Psychother Res.

[42] Government SA (2005). The children’s act no.38 of 2005.

[43] Puri MC, Maharjan DC, Dahal M (2023). Intimate partner violence, food insecurity and COVID-19 among newly married women in Nawalparasi district of Nepal: a longitudinal study. *Sex Reprod Health Matters*.

[44] Campbell L, Tan RKJ, Uhlich M (2023). Intimate Partner Violence During COVID-19 Restrictions: A Study of 30 Countries From the I-SHARE Consortium. J Interpers Violence.

[45] Peterman A, O’Donnell M (2020). COVID-19 and violence against women and children: a third research round up for the 16 days of activism.

[46] Liu J, Chai L, Zhu H (2023). COVID-19 impacts and adolescent suicide: The mediating roles of child abuse and mental health conditions. Child Abuse Negl.

[47] Arenas-Arroyo E, Fernandez-Kranz D, Nollenberger N (2021). Intimate partner violence under forced cohabitation and economic stress: Evidence from the COVID-19 pandemic. J Public Econ.

[48] Decker MR, Wood SN, Thomas HL (2022). Violence against women from partners and other household members during COVID-19 in Burkina Faso and Kenya. BMC Public Health.

[49] Saloshni N, Nithiseelan NR (2022). Vulnerability of South African women workers in the COVID-19 pandemic. Front Public Health.

[50] Arndt C, Davies R, Gabriel S (2020). Impact of covid-19 on the South African economy.

[51] (2020). Social relief of distress grant: Republic of South Africa. https://www.gov.za/services/social-benefits/social-relief-distress#:~:text=What%20do%20you%20get%3F,extended%20for%20another%20three%20months.

[52] Venter A, Lewis CM, Saffy P (2020). Locked down: Impact of COVID-19 restrictions on trauma presentations to the emergency department. S Afr Med J.

[53] Foran HM, O’Leary KD (2008). Alcohol and intimate partner violence: a meta-analytic review. Clin Psychol Rev.

[54] Testa M (2004). The role of substance use in male-to-female physical and sexual violence: a brief review and recommendations for future research. J Interpers Violence.

[55] Testa M, Quigley BM, Leonard KE (2003). Does alcohol make a difference? Within-participants comparison of incidents of partner violence. J Interpers Violence.

[56] Satyanarayana VA, Nattala P, Selvam S (2016). Integrated Cognitive Behavioral Intervention Reduces Intimate Partner Violence Among Alcohol Dependent Men, and Improves Mental Health Outcomes in their Spouses: A Clinic Based Randomized Controlled Trial from South India. J Subst Abuse Treat.

[57] O’Farrell TJ, Murphy CM, Stephan SH (2004). Partner violence before and after couples-based alcoholism treatment for male alcoholic patients: the role of treatment involvement and abstinence. J Consult Clin Psychol.

[58] Hartmann M, Appaiah P, Datta S (2024). “My Family Was Also Happy”: Couples’ Qualitative Reports of a Combined Behavioral Economics and Cognitive Behavioral Therapy Intervention to Reduce Alcohol Use and Intimate Partner Violence. Violence Against Women.

[59] Hartmann M, Datta S, Browne EN (2021). A Combined Behavioral Economics and Cognitive Behavioral Therapy Intervention to Reduce Alcohol Use and Intimate Partner Violence Among Couples in Bengaluru, India: Results of a Pilot Study. J Interpers Violence.

[60] Patel U, Roesch R (2022). The Prevalence of Technology-Facilitated Sexual Violence: A Meta-Analysis and Systematic Review. Trauma Violence Abuse.

[61] Mitchell M, Wood J, O’Neill T (2025). Technology-facilitated violence: A conceptual review. Criminology & Criminal Justice.

[62] Champion AR, Oswald F, Khera D (2022). Examining the Gendered Impacts of Technology-Facilitated Sexual Violence: A Mixed Methods Approach. Arch Sex Behav.

[63] Linares R, Aranda M, García-Domingo M (2021). Cyber-dating abuse in young adult couples: Relations with sexist attitudes and violence justification, smartphone usage and impulsivity. PLoS One.

[64] Champion A, Oswald F, Pedersen CL (2021). Technology-facilitated sexual violence and suicide risk: A serial mediation model investigating bullying, depression, perceived burdensomeness, and thwarted belongingness. Can J Hum Sex.

[65] Powell A, Henry N (2019). Technology-Facilitated Sexual Violence Victimization: Results From an Online Survey of Australian Adults. J Interpers Violence.

[66] Wandera N (2018). Technology-facilitated gender-based violence: what is it, and how do we measure it.

[67] Henry N, Powell A (2018). Technology-Facilitated Sexual Violence: A Literature Review of Empirical Research. Trauma Violence Abuse.

[68] Plan International (2020). Free to be online? Girls’ and young women’s experiences of online harassment.

[69] Hicks J (2021). Global Evidence on the Prevalence and Impact of Online Gender-based Violence (OGBV).

[70] Silver L, Smith A, Johnson C (2019). Mobile connectivity in emerging economies.

[71] Kreutzer T (2009). Generation mobile: online and digital media usage on mobile phones among low-income urban youth in South Africa, 30.

[72] Hensen B, Mackworth-Young CRS, Simwinga M (2021). Remote data collection for public health research in a COVID-19 era: ethical implications, challenges and opportunities. Health Policy Plan.

[73] Dutton MA, Holtzworth-Munroe A, Jouriles E (2003). Recruitment and retention in intimate partner violence research.

[74] Kidman R, Nachman S, Dietrich J (2018). Childhood adversity increases the risk of onward transmission from perinatal HIV-infected adolescents and youth in South Africa. Child Abuse Negl.

[75] Meinck F, Cluver LD, Boyes ME (2015). Risk and Protective Factors for Physical and Sexual Abuse of Children and Adolescents in Africa. Trauma Violence Abuse.

