# Supplementary material for: Mixed-method longitudinal investigation of sexual and gender-based violence following COVID-19 in South Africa
Source: BMJ Public Health. 2025 Apr 15;3(1):e001697. doi: 10.1136/bmjph-2024-001697 (PMC12010298; doi:10.1136/bmjph-2024-001697)
Supplement: online supplemental file 1 [file bmjph-3-1-s001.pdf]

Supplementary file 1. Quantitative survey items by type of violence and study visit

Domain	Baseline items	Month 3 and 6 items
**Household violence**		
Psychological violence	Since the lockdown started, have you witnessed a household or family member being victimized through verbal and/or emotional violence (e.g. humiliated, threatened)?	In the past 3 months, have you witnessed a household or family member being victimized through verbal and/or emotional violence (e.g. humiliated, threatened)?
Physical violence	Since the lockdown started, have you witnessed a household or family member being victimized through physical violence (e.g. pushed, kicked, slapped)?	In the past 3 months, have you witnessed a household or family member being victimized through physical violence (e.g. pushed, kicked, slapped)?
Sexual violence	Since the lockdown started, are you aware of a household or family member being forced to have sexual intercourse against their will?	In the past 3 months, are you aware of a household or family member being forced to have sexual intercourse against their will?
**Partner violence**		
Psychological violence	Did your partner do any of the following things to you during and/or before the lockdown:<br>[*asked separately*]<br>1. Tried to restrict (online or phone) contact with your family?<br>2. Insulted you or made you feel bad about yourself?<br>3. Threatened to hurt you or someone that you care about?	Did your partner do any of the following things to you in the past 3 months:<br>[*asked separately*]<br>1. Tried to restrict (online or phone) contact with your family?<br>2. Insulted you or made you feel bad about yourself?<br>3. Threatened to hurt you or someone that you care about?
Physical violence	4. Slapped, hit, kicked, dragged, pushed, shoved, choked or burnt you?	6. Slapped, hit, kicked, dragged, pushed, shoved, choked or burnt you?<br>Threatened to use or

	5. Threatened to use or actually used a knife or gun against you?	actually used a knife or gun against you?
Sexual violence	1. Physically forced you to have sexual intercourse when you did not want to?<br>2. Made you have sexual intercourse when you did not want to because you were afraid of what your partner might do?	3. Physically forced you to have sexual intercourse when you did not want to?<br>4. Made you have sexual intercourse when you did not want to because you were afraid of what your partner might do?
