# Supplementary material for: Mixed-method longitudinal investigation of sexual and gender-based violence following COVID-19 in South Africa
Source: BMJ Public Health. 2025 Apr 15;3(1):e001697. doi: 10.1136/bmjph-2024-001697 (PMC12010298; doi:10.1136/bmjph-2024-001697)
Supplement: online supplemental file 2 [file bmjph-3-1-s002.pdf]

Supplementary file 2. Model fit statistics

Shape	Parameter	BIC	BIC	Bayes Factor	AIC	LL	Entropy	Smallest group (%)	Smallest group (N)	Avg Posterior probability, per group			
	**Household SGBV**												
	**All**	N=1161	N=522										
	1	-621	-620.19		-615.93	-613.93							
	1 1	-605.92	-603.89		-593.24	-588.24	0.41	40%	208.80	0.83	0.78		
	1 1 1	-615.77	-612.53		-595.5	-587.5	0.6	10%	49.59	0.89	0.52	0.73	
	1 1 1 1	-626.34	-621.88		-598.46	-587.46	0.57	5%	27.67	0.78	0.68	*	0.59
	1 3	-605.46	-602.2		-587.76	-580.76	0.57	26%	135.77	0.91	0.77		
	2 1	-609.45	-607.02		-594.24	-588.24	0.40	40%	208.80	0.83	0.78		
	2 2	-609.58	-606.74		-591.84	-584.84	0.58	24%	125.28	0.91	0.73		
	2 3	-608.91	-605.65		-588.68	-580.68	0.58	24%	125.28	0.91	0.73		
	3 3	-616.65	-613.00		-593.84	-584.84	0.58	24%	125.28	0.91	0.73		
	**Females**	N=848	N=366										
	2 2	-436.12	-433.17		-419.52	-412.52	0.66	20%	73.2	0.91	0.83		
	**Males**	N=320	N=154										
	2 2	-185.24	-182.68		-172.05	-165.05	0.54	45%	69.3	0.80	0.99		
	**IPV**												
	**All**	N=572	N=341										
	1	-193.71	-193.2		-189.37	-187.37							
	1 1	-199.9	-198.61		-184.00	-184.30	0.61	10%	34.10	0.92	0.76		
	1 1 1	-208.47	-206.4		-191.08	-183.08	0.77	3%	8.53	0.94	0.66	0.89	
	1 1 1 1	-218	-215.15		-194.08	-183.08	0.38	3%	8.53	*	0.55	0.66	0.89
	1 3	-206.32	-204.51		-191.10	-184.10	0.51	14%	47.74	0.84	0.89		
	2 1	-202.44	-200.89		-189.39	-183.39	0.89	2%	6.82	0.98	0.79		
	2 2	-206.02	-204.21		-190.80	-183.80	0.73	8%	25.92	0.94	0.97		
	2 3	-209.20	-207.13		-191.80	-183.80	0.73	8%	25.92	0.94	0.97		
	3 3	-211.96	-209.63		-192.39	-183.39	0.89	2%	6.82	0.98	0.79		
	**Females**	N=409	N=239										
	2 2	-143.16	-141.28		-129.11	-122.11	0.73	8%	18.642	0.80	0.95		
	**Males**	N=159	N=100										
	2 2	-72.57	-70.95		-61.83	-54.83	0.93	2%	2	0.99	0.98		
	**IPV, coding non-partnered participants as 'no IPV'**												
	**All**	N=1198	N=531										
	2 2	-252.89	-250.05		-235.09	-228.09	0.94	1%	7.43	0.79	0.99		
	**Females**	N=862	N=370										
	2 2	-175.06	-172.10		-158.41	-151.41	0.48	16%	59.2	0.99	0.87		
	**Males**	N=330	N=159										
	2 2	-88.41	-85.85		-75.11	-68.11	0.99	1%	1.59	0.99	0.99		

Footnotes: Displays fit criteria used to assess the best model fit for the full sample and dissagregated by sex. The gray row indicates the best fit.
BIC=Bayesian Information Criteria; AIC=Akaike Information Criteria; LL=Log Liklihood; *Unable to calculate
