# Supplementary material for: Mixed-method longitudinal investigation of sexual and gender-based violence following COVID-19 in South Africa
Source: BMJ Public Health. 2025 Apr 15;3(1):e001697. doi: 10.1136/bmjph-2024-001697 (PMC12010298; doi:10.1136/bmjph-2024-001697)
Supplement: online supplemental file 3 [file bmjph-3-1-s003.docx]

**Supplementary file 3. SGBV exposure, overall and by trajectory groups by visit**

| **Baseline variables** | **Total (N=535)** | **Household SGBV trajectory group** | | | | | | **IPV trajectory group** | | | | | |
| --- | --- | --- | --- | --- | --- | --- | --- | --- | --- | --- | --- | --- | --- |
|  |  | **Group 1 – Consistently low (n=412)** | | | **Group 2 – Declining to moderate (n=123)** | | | **Group 1 – Consistently low (n=476)** | | | **Group 2 – Declining to moderate (n=68)** | | |
|  | **N (%)** | **n(%)** |  |  | **n (%)** |  |  | **n(%)** |  |  | **n (%)** |  |  |
|  |  | **Bl** | **M3** | **M6** | **Bl** | **M3** | **M6** | **Bl** | **M3** | **M6** | **Bl** | **M3** | **M6** |
| **Household violence** | | | | | | | | | | | | | |
| **Any HV** | 166 (32.8%) | 63 (16.1%) | 21 (6.4%) | 12 (6.9%) | 103 (90.4%) | 47 (45.6%) | 26 (40.0%) | 130 (29.1%) | 51 (13.6%) | 28 (13.8%) | 36 (60.0%) | 17 (31.5%) | 10 (27.8%) |
| Emotional HV | 121 (23.0%) | 42 (10.2%) | 14 (4.3%) | 8 (4.6%) | 79 (69.3%) | 32 (30.8%) | 22 (33.3%) | 95 (20.4%) | 34 (9.0%) | 21 (10.3%) | 26 (43.3%) | 12 (21.8%) | 9 (24.3%) |
| Physical HV | 104 (19.8%) | 38 (9.2%) | 11 (3.4%) | 8 (4.6%) | 66 (57.9%) | 32 (30.8%) | 19 (28.8%) | 78 (16.7%) | 33 (8.8%) | 21 (10.3%) | 26 (43.3%) | 10 (18.2%) | 6 (16.2%) |
| Sexual HV | 17 (3.2%) | 7 (1.7%) | 2 (0.6%) | 1 (0.6%) | 10 (8.8%) | 3 (2.9%) | 4 (6.2%) | 12 (2.6%) | 3 (0.8%) | 3 (1.5%) | 5 (8.3%) | 2 (3.6%) | 2 (5.4%) |
| **Intimate partner violence** | | | | | | | | | | | | | |
| **Any IPV** | 36 (13.7%) | 18 (9.0%) | 5 (3.4%) | 2 (3%) | 18 (29.0%) | 9 (17.0%) | 7 (22%) | 15 (6.8%) | 6 (3.4%) | 2 (2%) | 21 (51.2%) | 8 (30.8%) | 7 (37%) |
| Emotional IPV | 6 (2.3%) | 2 (1.0%) | 2 (1.4%) | 1 (1%) | 4 (6.5%) | 2 (3.8%) | 1 (1%) | 4 (1.8%) | 2 (1.1%) | 1 (1%) | 2 (4.9%) | 2 (7.7%) | 1 (5%) |
| Physical IPV | 22 (8.4%) | 11 (5.5%) | 2 (1.4%) | 1 (1%) | 11 (17.7%) | 7 (13.2%) | 4 (12%) | 10 (4.5%) | 3 (1.7%) | 1 (1%) | 12 (29.3%) | 6 (23.1%) | 4 (21%) |
| Sexual IPV | 13 (5.0%) | 5 (2.5%) | 1 (0.7%) | 2 (3.8%) | 8 (12.9%) | 1 (1%) | 1 (3%) | 2 (0.9%) | 1 (0.6%) | 1 (1%) | 11 (26.8%) | 2 (7.7%) | 1 (5%) |

SGBV = sexual and gender-based violence; IPV = intimate partner violence; HV = household violence. Note that sub-categories of violence are not mutually exclusive.

Trajectory groups models were adjusted for gender, HIV status, household composition (no. of people in the household), food insecurity, and probable CMD.
